# Repair of Penetrating Pericardial and Diaphragmatic Injury with Cormatrix® Patch in a Case of Suicide Attempt

**DOI:** 10.21470/1678-9741-2016-0055

**Published:** 2017

**Authors:** Federica Jiritano, Carlo Garrasi, Lucia Cristodoro, Egidio Bevacqua, Pasquale Mastroroberto

**Affiliations:** 1 Cardiac Surgery Unit, Magna Graecia University of Catanzaro, Italy.

**Keywords:** Diaphragm/Injuries, Pericardium/Injuries, Thoracic Injuries/Surgery, Suicide, Attempted

## Abstract

The authors report the case of a suicide attempt. A 59-year-old man with self-inflicted penetrating chest trauma underwent emergency cardiothoracic surgery. Pre-operative computed tomography scan showed critical proximity between the blade and the right ventricle. Intraoperative findings showed a pericardial laceration and a huge diaphragmatic lesion with heart and abdominal organs integrity. The diaphragm muscle was repaired with a CorMatrix® patch, an acceptable alternative to the traditional synthetic mesh avoiding infection and repeated herniation.

**Table:** 

Abbreviations, acronyms & symbols	
CT DI	= Computed tomography = Diaphragmatic Injuries

## INTRODUCTION

Diaphragmatic injuries (DI) are a diagnostic and therapeutic challenge for the surgeon. It is a quite rare trauma, with only 4-5% of patients undergoing surgery for DI. The main cause of DI are blunt trauma of the chest and abdomen (75%), whereas, more rarely, DI are due by penetrating trauma (25%)^[1]^. Clinical presentations are different among patients: they can range from unstable hemodynamic condition linked to active bleeding from organs and diaphragm, to dyspnea and intestinal obstruction due to visceral herniation of abdominal organs in the thoracic cavity. In this case report, the diaphragmatic injury could not go unnoticed because the patient was admitted with a knife still in place, following a suicide attempt.

## CASE REPORT

**Fig. 1:**
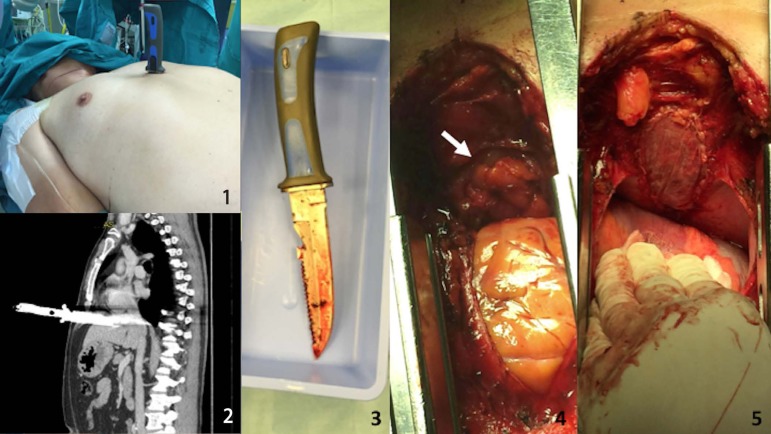
1 - Knife penetrating the chest; 2- preoperative CT-scan; 3 - knife removed; 4 - mediastinal view - the arrow shows omentum herniation through the diaphragmatic injury; 5 - CorMatrix® patch closing the diaphragmatic rupture.

A 59-year-old man with self-inflicted penetrating chest trauma was admitted in our hospital. He tried before to commit suicide hanging himself with a rope, as the signs on his neck revealed. The patient arrived on mechanical ventilation. His blood pressure was 90/50 mmHg, the heartbeat was 92 bpm and haemoglobin was 10.8 mg/dl. On clinical examination, the patient had still the knife into his body (Figure 1-1) with whole blade inflicted just below the xiphoidal process, leaving outside only the knife handle. Pre-operative computed tomography (CT) scan showed critical proximity between the knife blade and the right ventricle (Figure 1-2) so suspecting a right ventricle injury arterial femoral cannulation for cardiopulmonary bypass was achieved. A median sternotomy was performed, the pericardium was longitudinally opened draining about of 500 ml of haematic fluid and the knife was safely removed (Figure 1-3). Intraoperative findings showed pericardial and diaphragmatic lesions with heart and abdominal organs integrity (Figure 1-4). The diaphragmatic lesion showed an active bleeding into the mediastinum. A direct suture of the diaphragm muscle was not possible because of the huge laceration and the friable edges so the diaphragm muscle was repaired with a CorMatrix® patch (Figure 1-5). Chest drain tubes were placed and the sternum was closed with interrupted steel wires. Despite the diaphragmatic surface has been increased in order to repair the muscle itself, patient’s gas exchanges were not affected. The patient, in fact, was extubated the day after surgery. The postoperative course was uneventful and the patient was discharged on the 5^th^ postoperative day, after a psychiatric consulting.

## DISCUSSION

The diaphragm is the main respiratory muscle and it has the most significant function in respiratory excursion, carrying more than 70% of the work. Moreover, it has the ability to maintain the negative thoracic pressure necessary for respiratory mechanics and to promote the venous return to the heart. The diaphragm muscle separates the chest from the abdomen, two cavities with different pressure inside. Any lesion of this muscle causes a possible migration of the abdominal organs in the thorax. This phenomenon could lead to respiratory failure and cardiovascular collapse, due to alterations of the respiratory mechanics and the venous return. Although diaphragmatic perforation caused by stab wounds are rare, they could cause the herniation of the abdominal contents into the thoracic cavity leading to life-threatening complications^[2]^. Its diagnosis is difficult despite the continuous development in radiological and echographic fields. Diaphragmatic injury may present acutely with haemodynamic and respiratory compromise and be associated with significant lesions to other organs or may not be diagnosed at the initial trauma at all and present later as a diaphragmatic hernia. In our case the diaphragmatic injury could not go unnoticed because the patient was admitted with a knife still in place, following a suicide attempt. In literature, the first report of diaphragmatic trauma dates back to 1853, when Bowditch^[2]^ described a diaphragmatic injury, in a dead victim of a gunshot penetrating trauma, during the autopsy. The first repair with positive outcomes of a penetrating diaphragmatic injury was described by Riolfi, in 1886^[3]^. Preoperative diagnosis of diaphragmatic injury is still a diagnostic challenge. The high mortality is also caused by the difficulty of studying this anatomical site in emergency conditions. Historically, CT-scan showed poor visualization of the diaphragm due to motion of the muscle itself. However, the advent of multiphasic spiral CT has led to a sensitivity of 80% and a specificity of 90%. Diaphragmatic injuries include wounds and diaphragm ruptures, due to a thoraco-abdominal blunt or penetrating traumas. The surgical treatment is controversial, particularly for the surgical approach and techniques.

The choice of surgical approach is debated. Laparoscopy and thoracoscopy are now the diagnostic and therapeutic choices. Laparoscopy allows assessment and repair of the diaphragm. The thoracoscopic approach is probably more useful in obvious thoracic injuries and in right-sided penetrating thoracoabdominal injuries. Surgical treatment consists of hernia reduction, pleural drainage and repair of diaphragmatic defect. Most diaphragmatic defects may be repaired primarily, especially in the acute setting due to the pliability of the diaphragm. However, for larger defects and patients with a delayed presentation, the diaphragmatic defect may be too large to repair primarily or the edges have become too thin and weak to hold suture. The stress of the continued use of the diaphragm during breathing, coughing, Valsalva, and even during cardiac motion are reasons enough for the use of a mesh repair for any large diaphragmatic defect or rupture. In cases of diaphragmatic disruption due to massive trauma, prosthetic non-absorbable mesh material is used to reconstruct the diaphragm. Synthetic mesh is a durable, cost-effective prosthesis that has been used for decades. However, an increasing amount of data is emerging regarding the complications of synthetic mesh repair, such as adhesion formation, erosion into surrounding structures, infections and need for subsequent explantation. This has led to an increase interest in the potential use of other patches for this purpose. Biological scaffold materials composed of extracellular matrix have already been shown to facilitate the constructive remodelling of many different tissues^[4]^. The extracellular matrix scaffold materials are derived from a variety of tissues, including heart valves, nerves, skeletal muscle and tendons. The mechanisms by which biological scaffold materials promote site-appropriate tissue reconstruction are not yet well understood. A number of different products available in the market are used in reconstructive surgery for various tissues and organs. In our case, we have used the CorMatrix® patch consisting of material made from small intestinal submucosa derived from pig jejunum and has been already used as an acellular biological scaffold in many different surgical applications^[5]^. In its natural form, the CorMatrix® patch consists primarily of several types of collagens, with smaller, but significant, amounts of glycosaminoglycans, glycoproteins and growth factors. CorMatrix® scaffold is composed also by proteoglycans and other non-fibrillar support structures (such as hyaluronic acid), which are very important for the healing processes. Hyaluronic acid is thought to help regulate the matrix density and inhibit scar formation during the healing process. Human endothelial and smooth muscle cells have been grown in vitro on hyaluronic acid biomaterial constructs with the objective to develop a new tissue-engineered vascular substitute^[6]^. It is more resistant to infection and may be placed in infected fields with minimal risk of infection compared to synthetic mesh. Trauma patients with blunt diaphragmatic rupture often have a concomitant bowel or lung injury with contamination. Then, CorMatrix® scaffold can be placed in these hostile environments with less risk of infection when compared to synthetic mesh. Using this type of material to reinforce a structure that continually moves like the diaphragm could be advantageous in that the diaphragm can repair itself via normal biologic pathways. Synthetic inert materials without the ability to catalyze regeneration and incorporation into dynamic native tissues may be subjected to fatigue stress of the material and suture due to the constant movement of the diaphragm.

## CONCLUSION

In conclusion, this case could be considered as a very rare form of suicide attempt with double distinctive elements: “self-made hanging” and “self-inflicted penetrating chest trauma” by a large knife used for diving activities. In order to repair diaphragmatic lesion, the use of CorMatrix® patch may be considered as an acceptable alternative to the traditional synthetic mesh avoiding infection and repeated herniation.

**Table:** 

Authors’ roles & responsibilities	
FJ	Conception and study design; realization of operations; manuscript redaction or critical review of its content; final manuscript approval
CG	Realization of operations; manuscript approval
LC	Realization of operations; manuscript approval
EB	Realization of operations; manuscript approval
PM	Conception and study design; realization of operations; manuscript redaction or critical review of its content; final manuscript approval
